# The safety attitudes questionnaire in Chinese: psychometric properties and benchmarking data of the safety culture in Beijing hospitals

**DOI:** 10.1186/s12913-017-2543-2

**Published:** 2017-08-23

**Authors:** Ying Cui, Xiuming Xi, Jinsheng Zhang, Jiang Feng, Xiaoxiao Deng, Ang Li, Jianxin Zhou

**Affiliations:** 1 Fu Xing Hospital, Capital Medical University, Beijing, China; 2 Beijing Friendship Hospital, Capital Medical University, Beijing, China; 3 Beijing Tian Tan Hospital, Capital Medical University, Beijing, China

**Keywords:** Patient safety, Safety culture, Safety attitudes questionnaire, Cross-sectional survey, Reliability, Validity

## Abstract

**Background:**

In China, increasing attention has been devoted to the patient safety culture within health administrative departments and healthcare organizations. However, no official version of a patient safety culture assessment tool has been published or is widely used, and little is known about the status of the safety culture in Chinese hospitals. The aims of this study were to examine the reliability and validity of the Safety Attitudes Questionnaire in Chinese and to establish benchmark data on the safety culture in Beijing.

**Methods:**

Across-sectional survey on patient safety culture was conducted from August to October 2014 using the Safety Attitudes Questionnaire in Chinese. Using a stratified random sampling method, we investigated departments from five integrative teaching hospitals in Beijing; frontline healthcare workers in each unit participated in the survey on a voluntary basis. The internal consistency and reliability were tested via Cronbach’s alpha, and the structural validity of the questionnaire was tested using a correlation analysis and confirmatory factor analysis. The patient safety culture in the five hospitals was assessed and analyzed.

**Results:**

A total of 1663 valid questionnaires were returned, for a response rate of 87.9%. Cronbach’s alpha of the total scale was 0.945, and Cronbach’s alpha for the six dimensions ranged from 0.785 to 0.899. The goodness-of-fit indices in the confirmatory factor analysis showed an acceptable but not ideal model fit. The safety attitude score of healthcare workers in the five hospitals was 69.72, and the positive response rate was 38.57% overall. The positive response rates of the six dimensions were between 20.80% and 59.31%.

**Conclusions:**

The Safety Attitudes Questionnaire in Chinese has good internal consistency, and the structural validity and reliability are acceptable. This questionnaire can be used to assess the safety culture in Beijing hospitals, but some items require further refinement. The patient safety culture in Beijing hospitals must be improved in certain key areas.

**Electronic supplementary material:**

The online version of this article (doi:10.1186/s12913-017-2543-2) contains supplementary material, which is available to authorized users.

## Background

In 1999, the Institute of Medicine published a report entitled “To err is human: Building a safety health system” and noted that *“health care organizations must develop a ‘culture of safety’ such that their workforce and processes are focused on improvement of the reliability and safety of care for patients”* [1]. Since then, as one of these patient safety promotion projects, safety culture has received attention from medical and health organizations worldwide [1–3].

China is in the beginning stages of establishing a patient safety culture. A patient safety event reporting system was established in hospitals as part of the risk management system by the China Hospital Association in 2007, and "Building a culture of patient safety" was proposed in 2014 as one of the top 10 patient safety goals. The National Health Administration Department has committed to improving medical quality and ensuring patient safety by launching various nationwide activities, such as the “Medical Quality Tour”, “Three Good and One Satisfaction”, and “the National Healthcare Improvement Initiative”, and has made substantial progress.

The WHO definition of a safety culture is as follows: *“The safety culture of an organization is the product of individual and group values, attitudes, perception, competencies and patterns of behavior that determine the commitment to, and the style and proficiency of, an organization’s health and safety management”* [4]. Studies have shown that a positive safety culture can promote safety behavior among medical personnel, reduce the occurrence of adverse medical events, reduce the patient readmission rate, and decrease hospitalization time [5–8].

A number of safety culture assessment tools have been developed in the healthcare field [9–12], and they can be used to assess the safety culture to identify areas for improvement, raise awareness about patient safety, evaluate patient safety interventions or programs, track changes over time, conduct internal and external benchmarking, and fulfill directives or regulatory requirements [3].

According to a systematic literature review of the safety culture tools currently in use, the Safety Attitudes Questionnaire (SAQ) was found to exhibit superior characteristics in 24 of 25 indicators. The SAQ is one of “two tools that have been used to evaluate the associations between patient safety climate scores and process measures theorized to be associated with improved patient outcomes”, and “only the SAQ has been used to explore the relationship between safety climate scores and patient outcomes” [13]. The SAQ is a psychometrically sound tool that has been tested on a large sample [9]. It has been translated into different languages and used in various countries, including the United States [9], the United Kingdom [9], New Zealand [9], Sweden [14, 15], Norway [16], Australia [17], Italy [18], Turkey [19], and Chinese Taiwan [20]. The SAQ demonstrated good reliability and validity in these studies, and the survey findings could provide a baseline database of safety culture assessments and indicate the strengths and weaknesses at the clinical level. Regular safety culture assessments have been performed by some health administration nongovernmental organizations [20, 21] to conduct a comparative analysis, and continuous efforts have been initiated to minimize the variations in safety culture among hospitals.

However, patient safety culture and its assessment tools are still being researched in China. Due to the linguistic differences between Chinese and Western cultures, the introduction and modification of the scale and its validity and effectiveness have not yet been recognized by the healthcare industry. Knowledge of the psychometric properties of the SAQ and of benchmarking data in China is limited. This study aims to test the applicability of the SAQ in Chinese healthcare organizations in Beijing, to investigate the current status of the patient safety culture, to identify the advantages and disadvantages, and to provide basic information for the improvement of the patient safety culture in China.

## Methods

### Survey instrument

The SAQ was developed by the University of Texas [9]; 25% of the items are from the Flight Management Attitude Questionnaire (FMAQ) [22, 23], and 75% of the items pertain to healthcare industry characteristics [24, 25]. The SAQ contains six dimensions: Teamwork Climate, Safety Climate, Job Satisfaction, Stress Recognition, Perception of Management, and Working Conditions. It has been adapted for use in different settings; these alternate versions include the ICU version, Operating Room version, Ambulatory version, Labor & Delivery version, Pharmacy version, and Short-Form version. The item content is the same for each version of the SAQ, with minor modifications to reflect the clinical area. The SAQ uses a five-point Likert scale: 1 = Strongly Disagree, 2 = Slightly Disagree, 3 = Neutral, 4 = Slightly Agree, 5 = Strongly Agree.

The present research adopted the generic SAQ Short-Form version (available at https://med.uth.edu/chqs/surveys/safety-attitudes-and-safety-climate-questionnaire/). This version consists of 36 items, 31 of which are categorized into 6 dimensions and 5 of which do not belong to any dimension. The items are as follows: Teamwork Climate: Items 1–6; Safety Climate: Items 7–13; Job Satisfaction: Items 15–19; Stress Recognition: Items 20–23; Perceptions of Management: Items 24–29 (each of these items is measured at two levels–unit and hospital); and Working Conditions: Items 30–32. Items 14 and 33–36 are not part of the subscales above; Items 2, 11, 20–23 and 36 are reverse-scored [9].

### Translation and adaptation of the SAQ

The translation of the SAQ Short-Form version into Chinese was performed independently by two staff members. The comparison and modification of the translation with reference to the SAQ Taiwan version was conducted by a clinical expert and a psychology expert with experience in medical English translation. Scientific and cultural adjustments were completed through two rounds of an expert seminar (focus group), including 15 experts in hospital management, health statistics, psychology, clinical medical care, and nursing. We organized the group discussions and the interviews of healthcare staff subjects in different positions while fully considering the equivalence and understandability between the Chinese version and the generic English version. The Safety Attitudes Questionnaire in Chinese see Additional file 1.

Based on the original scale, the social-demographic characteristics section was self-designed and included gender, age (years), education degree, profession, technical titles, administrative post, years working in the current department, and related information.

### Administration of the survey

Five integrative teaching hospitals were selected from affiliated or teaching hospitals of Capital Medical University in Beijing, including three tertiary-level hospitals and two secondary-level hospitals; the leaders of these hospitals expressed their willingness to participate in the survey. Using stratified random sampling, we selected the investigative departments from the five hospitals, including clinical departments (internal medicine, general surgery, and obstetrics and gynecology) and medico-technical departments (pharmacies, laboratories, radiology departments, etc.), while three special units (intensive care units, operating rooms, emergency rooms) were included due to the high rates of error with serious consequences [1]. The frontline healthcare workers in the investigation departments of the five hospitals participated in the survey on a voluntary basis. The inclusion criteria required that the respondents had worked in the department for at least 4 weeks and either influenced or were influenced by the “working environment” in the specific clinical area [9, 20].

From August to October 2014, the survey was conducted in five hospitals by the investigators using concentrated dissemination and processing on the spot in the selected departments. Based on the unified training, the investigators should have understood the purpose of the research, the connotations of safety culture and the meaning of each item in the SAQ. During the investigation, the investigators introduced the study’s background and purpose and clarified matters requiring attention to ensure thorough understanding among the respondents, encourage their compliance to avoid low recovery rates and reduce missing items. The questionnaire took approximately 10 to 15 min to complete. To ensure anonymity and confidentiality, the questionnaire was folded in half and directly handed to the investigators after completion.

Incomplete or invalid questionnaires were excluded, including those with more than 5 questions with missing answers, answers with obvious reaction tendencies (such as the use of extreme answers for questions, choosing the same answer for 10 questions in a row, or answering with a specific regularity) and those with the same answers.

### Data management and analysis

The data were read using Epidata software version3.1 and analyzed using SPSS19.0 for Windows; confirmatory factor analysis was conducted using Amos17.0. Negatively worded items were reverse-scored to ensure that their valence matched the positively worded items. To clarify the interpretation of the data, the five-point scale was converted to a 100-point scale, 1 → 0, 2 → 25, 3 → 50, 4 → 75, 5 → 100 [14, 18, 20, 26].

#### Reliability analysis

Thirty-one items in six dimensions were considered in the reliability and validity analysis. The reliability evaluation was used to measure the reliability, stability and consistency of the results of the questionnaire, that is, the size of the variance of the measurement value caused by random errors in the measurement process. As the reliability index, Cronbach’s alpha was used to evaluate the internal reliability of the questionnaire [27]. In the analysis of the split-half reliability, the questionnaire was divided into two halves, the correlation coefficient of the two halves of the score was calculated, and the reliability of the scale was estimated. The split-half reliability is linked to the internal consistency coefficient and was used to evaluate the consistency between the scores of two items. If the alpha coefficient was ≥0.70, the internal consistency reliability was considered acceptable [28], i.e., the cutoff = 0.70 [29], and when the coefficient was ≥0.8, the scale reliability was considered very good [30]. Because the medical staff was busy at work, this study did not retest the reliability assessment.

#### Validity analysis

The validity assessment provides an evaluation of the accuracy, validity and correctness of the scale, that is, the deviation between the measured value and the real value of the target construct. Content validity is the appropriateness and representativeness between the content and the subject of the scale, namely, whether the content of the reaction to the psychological characteristics of the measurement can measure or assess behavioral constructs. The assessment was generally rated by the expert group with a two-way breakdown evaluation: Very relevant = 4; Somewhat relevant = 3; Weakly relevant = 2; Not related = 1. The content validity index at the item level (I-CVI) was determined as part of the expert group according to the correlation of each item and the research concept. The number of experts whose scores were 3 or 4 was divided by the total number of experts to determine the I-CVI. When the number of experts is less than or equal to 5, the opinions of all experts must be consistent to ensure the validity of content; that is, the I-CVI must be 1. When the number of experts increases, this standard can be reduced, but the I-CVI must be higher than 0.78 [31]. The I-CVI of each item can be used to determine whether to retain, modify, or discard the scale. The content validity index at the scale level (S-CVI) is the proportion of the items that all experts rated 3 or 4, that is, the total number of the items all experts rated 3 or 4 divided by the total number of the items in the scale, and the S-CVI should be at least 0.80 [32]. In this study, the expert group was composed of six associate senior professors in clinical medical, hospital management, health statistics, or psychology.

Construct validity is used to evaluate whether the structure of the scale is consistent with its theoretical assumptions and whether the internal components of the measurement results are consistent with the designers’ intention to measure the field.

A correlation analysis is used to evaluate the construct validity of the measurement tool, including the correlation analysis between each dimension and item. Construct reliability (CR) values larger than 0.7 suggest good reliability, and values between 0.6 and 0.7 are potentially acceptable provided that other indicators of the model’s construct validity are good. High construct reliability indicates the existence of internal consistency [33].

A confirmatory factor analysis (CFA) was used to explore whether the factor structure of the scale model fit the actual data collected. A structural equation model was constructed to explore whether the index variable could be effectively used as a measure of the factors. The missing value was replaced by the average near points in the CFA but maintained null values in other statistical analyses [34].

The following indices were specifically considered: (i) the chi-square goodness-of-fit (χ^2^): the model was acceptable if the *p*-value of the chi-square test was not significant; (ii) normalized chi-square (NC), which is the chi-square divided by the degree of freedom (d.f.): a ratio ranging from 3 to 1 was acceptable for a model fit [35]; (iii) goodness-of-fit index (GFI), adjusted goodness-of-fit index (AGFI), the comparative fit index (CFI): a value within 0.90–1.00 was acceptable for indicating model fit [36]; and (iv) root mean square error of approximation (RMSEA): a value of approximately 0.05 or less indicated a close fit of the model in relation to the d.f. [37].

#### Benchmarking climate

Once the structure was confirmed, the mean scores of the safety factors for each respondent were calculated to determine the percentage of respondents with positive attitudes toward each safety dimension.

A positive response meant choosing the answers “agree slightly” or “agree strongly”, which indicated that the score was 75 or higher. The positive response rate of the dimension (or the item) = the number of scores ≥75 in the dimension (or the item) / the number of effective respondents. Dimensions (or items) with a positive response rate of 80% or higher were advantageous areas, and those lower than 60% were the improvement areas [14, 18, 20, 26].

## Results

Of the 1892 distributed questionnaires, 1663 effective questionnaires were collected; the effective rate was 87.90%. The loss rates for the 36 items were between 0.06% and 1.38%.

### Reliability of the SAQ in Chinese

Table 1 shows that the total Cronbach’s alpha was 0.945, and the Cronbach’s alpha values of the six dimensions were between 0.785 and 0.899. The split-half reliability of the scale was 0.869, the correlation coefficient between two parts was 0.769, and the split-half reliability indices of six dimensions were between 0.738 and 0.912. The SAQ in Chinese thus had high internal consistency.

**Table 1:** Cronbach’s α of the SAQ in Chinese

Dimension	Cronbach’s α	Split-half reliability Spearman-Brown coefficient
Teamwork climate	0.785	0.773
Safety climate	0.822	0.784
Job satisfaction	0.899	0.912
Stress recognition	0.881	0.844
Recognition of management	0.879	0.868
Working condition	0.785	0.738
Total Scale	0.945	0.869

### Validity of the SAQ in Chinese

In the CVI, the I-CVI of each item was between 0.8 and 1.0, higher than 0.78; the S-CVI was 0.86, which is greater than 0.80.

In the Pearson correlation test, the correlation coefficient between Stress Recognition and the entire questionnaire was low (*r* = 0.412); the correlation coefficients between the five dimensions and the entire questionnaire were higher (*r* = 0.617 to 0.874) and were also higher than the correlation coefficients between each dimension. The correlation coefficient between the Stress Recognition dimension and the other five dimensions was lower, between 0.174 and 0.196; the correlation coefficient between the remaining dimensions was between 0.572 and 0.777. The correlation coefficient between each dimension in the SAQ is shown in Table 2. The correlation coefficients between each item and its corresponding dimensions were higher than the correlation with the other dimensions (see Table 3). These findings indicate that the scale had good internal consistency.

**Table 2:** The correlation coefficient between each dimension of the SAQ in Chinese

Factor	Total scale	Teamwork climate	Safety climate	Job satisfaction	Stress recognition	Recognition of management	Working condition
Teamwork climate	0.837	1.000	…	…	…	…	…
Safety climate	0.874	0.777	1.000	…	…	…	…
Job satisfaction	0.850	0.681	0.707	1.000	…	…	…
Stress recognition	0.412	0.177	0.181	0.196	1.000	…	…
Recognition of management	0.860	0.678	0.720	0.728	0.174	1.000	…
Working condition	0.801	0.572	0.648	0.665	0.195	0.749	1.000

**Table 3:** The correlation coefficient between each item and dimension of the SAQ in Chinese

Dimension	Teamwork climate	Safety climate	Job satisfaction	Stress recognition	Recognition of management	Working condition
item						
TC 1	0.713	0.541	0.527	0.102	0.576	0.475
TC 2	0.535	0.334	0.243	0.176	0.229	0.188
TC 3	0.745	0.610	0.555	0.142	0.544	0.458
TC 4	0.719	0.563	0.455	0.116	0.484	0.430
TC 5	0.744	0.604	0.473	0.078	0.478	0.399
TC 6	0.753	0.640	0.636	0.113	0.559	0.474
SC 1	0.576	0.705	0.501	0.140	0.512	0.462
SC 2	0.644	0.751	0.542	0.141	0.546	0.476
SC 3	0.562	0.757	0.498	0.124	0.554	0.488
SC 4	0.599	0.742	0.605	0.107	0.599	0.539
SC 5	0.394	0.515	0.269	0.214	0.267	0.228
SC 6	0.488	0.698	0.482	0.058	0.498	0.475
SC 7	0.569	0.749	0.590	0.094	0.573	0.527
JS 1	0.443	0.466	0.743	0.184	0.507	0.487
JS 2	0.642	0.632	0.837	0.153	0.621	0.536
JS 3	0.604	0.631	0.881	0.133	0.648	0.577
JS 4	0.571	0.610	0.900	0.177	0.633	0.600
JS 5	0.619	0.646	0.859	0.182	0.665	0.604
SR 1	0.131	0.139	0.135	0.815	0.132	0.165
SR 2	0.115	0.111	0.154	0.890	0.132	0.152
SR 3	0.173	0.180	0.182	0.874	0.167	0.177
SR 4	0.186	0.190	0.203	0.855	0.165	0.174
RM 1	0.601	0.632	0.654	0.098	0.838	0.655
RM 2	0.448	0.473	0.430	0.138	0.712	0.455
RM 3	0.634	0.652	0.683	0.175	0.877	0.685
RM 4	0.526	0.571	0.584	0.144	0.837	0.606
RM 5	0.577	0.632	0.643	0.159	0.845	0.679
WC 1	0.362	0.421	0.475	0.166	0.509	0.776
WC 2	0.422	0.492	0.492	0.170	0.598	0.794
WC 3	0.500	0.553	0.574	0.122	0.630	0.800
WC 4	0.569	0.625	0.585	0.150	0.665	0.794

*Note*: TC 1–6 indicates the six items in Teamwork climate. SC1–7 indicates the seven items in Safety climate. JS 1–7 indicates the seven items in Job satisfaction. SR 1–4 indicates the four items in Stress recognition. RM 1–5 indicates the five items in Recognition of management. WC 1–4 indicates the four items in Working condition

**Fig. 1:**
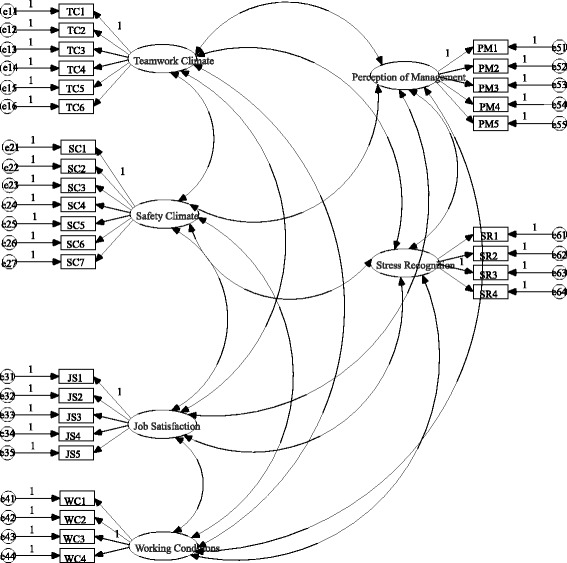
The structural equation model

In the CFA, a structural equation model was constructed (see Fig. 1). Influenced by the large sample size, the χ^2^ value and NC value were greater, χ^2^ = 1410.544(*p* = 0.000), NC = 3.474. However, considering the other goodness-of-fit statistics, i.e., GFI = 0.948, AGFI = 0.936, CFI = 0.963, RMSEA = 0.039 (Table 4), the GFI, AGFI and CFI were greater than 0.90, and the RMSEA value was less than 0.05; therefore, the construct validity was not ideal but was acceptable. Goodness-of-fit indices for the model are shown in Table 4.

**Table 4:** Goodness-of-fit indices for the model

Statistical test	Actual fitting values	Adaptation of the criteria or the critical value	Result
Absolute fit indices			
χ^2^	1,410.544 (*p* = 0.000)	*p* > 0.05	unsatisfactory
GFI	0.948	>0.90	satisfactory
AGFI	0.936	>0.90	satisfactory
RMR	0.060	<0.05	unsatisfactory
RMSEA	0.039	<0.05	satisfactory
NCP	1,044.544	The less the better	unsatisfactory
ECVI	0.957,0.597,18.410	Default model less than Independence model and Saturated model	unsatisfactory
Relative fit indices			
NFI	0.954	>0.90	satisfactory
RFI	0.947	>0.90	satisfactory
IFI	0.967	>0.90	satisfactory
TLI(NNFI)	0.962	>0.90	satisfactory
CFl	0.963	>0.90	satisfactory
Parsimonious fit indices			
PGFI	0.776	>0.05	satisfactory
PNFI	0.833	>0.05	satisfactory
CN	535	>200	satisfactory
NC	3.474	<3	unsatisfactory
AIC	1,590.544; 992.000; 30,597.421	Default model less than Independence model and Saturated model	unsatisfactory
CAIC	2,168.018; 4,174.524; 30,796.329	Default model less than Independence model and Saturated model	satisfactory

*Note*: *GFI* Goodness-of-fit index, *AGFI* Adjusted goodness-of-fit index, *RMR* Root mean square residual, *RMSEA* Root mean square error of approximation, *NCP* Non-centrality parameter, *ECVI* Expected cross-validation index, *NFI* Normed fit index, *RFI* Relative fit index, *IFI* Incremental fit index, *TLI* Tacker-Lewis index, *CFI* Comparative fit index, *PGFI* Parsimony Goodness-of-Fit Index, *PNFI* Parsimonious Normed Fit Index, *CN* Critical N, *NC* Normalized chi-square, *AIC* Akaike information criteria, *CAIC* The Consistent Version of AIC

### Demographic information

Of the 1663 respondents, 652 were in a secondary-level hospital (39.21%) and 1011 were in a tertiary-level hospital (60.79%). A total of 78.00% of the respondents were in clinical departments, 15.45% were in a medico-technical department, and 6.55% were in an outpatient department. The majority of the respondents were female (79.38%), and 76.47% were young (ages 21–40 years). More than half were nurses (55.06%), followed by physicians (30.69%), technicians (6.91%), and pharmacy personnel (6.25%). The demographics of the respondents are provided in Table 5.

**Table 5:** Characteristics of the safety attitude survey respondents

Characteristics	Valid frequency	Percent(%)
Hospital level		
Secondary-level hospitals	652	39.21
Tertiary-level hospitals	1011	60.79
Department category		
Obstetrics and gynecology department	112	7.33
Intensive care units	114	7.47
Operating rooms	119	7.79
The wards of internal medicine	406	26.59
General surgery	340	22.27
Emergency rooms	105	6.88
Outpatient department	100	6.55
Medico-technical departments	231	15.13
Gender		
Male	338	20.62
Female	1301	79.38
Age groups(years)		
≤ 20	14	0.85
21 ~ 30	743	45.06
31 ~ 40	518	31.41
41 ~ 50	294	17.83
≥ 51	80	4.85
Education degree		
Senior high schools and middle special schools	81	4.90
College degree	626	37.85
Bachelor degree	696	42.08
Master degree	220	13.30
Doctor degree	31	1.87
Job discipline		
Physicians	506	30.69
Nurses	908	55.06
Pharmacists	103	6.25
Technicians	114	6.91
Others	18	1.09
Technical titles		
None	178	10.77
Primary title	791	47.88
Middle title	541	32.75
Associated advanced title	103	6.23
Advanced title	39	2.36
Administrative post		
Director of the department	44	2.68
Nursing manager	58	3.53
Normal staff	1511	92.02
Others	29	1.77
Years working in the current department		
< 6 months	182	11.06
6 ~ 11 months	68	4.13
1 ~ 2 years	228	13.86
3 ~ 7 years	510	31.00
8 ~ 12 years	284	17.26
13 ~ 20 years	215	13.07
≥ 21 years	158	9.60
Have participated in the survey before		
Yes	447	27.01
No	1208	72.99

### Benchmarking climate

Table 6 shows that the mean score for the healthcare workers in five hospitals was 69.72 (SD = 15.47). The six dimensions from high to low scores were as follows: Teamwork Climate: 74.87 (SD = 18.23); Safety Climate: 73.82 (SD = 17.51); Job Satisfaction: 72.43 (SD = 22.50); Recognition of Management: 69.64 (SD = 19.68); Working Conditions: 68.59 (SD = 20.09); and Stress Recognition: 44.53 (SD = 28.70). Stress Recognition showed the greatest variability, and Safety Climate showed the least variability. The positive response rate of the healthcare workers in the five hospitals was 38.57%; the positive response rates of the six dimensions were from 20.80% to 59.31%. From high to low, these rates were as follows: Teamwork Climate: 59.31%; Safety Climate: 54.09%; Job Satisfaction: 54.63%; Working Conditions: 46.69%; Recognition of Management: 45.97%; and Stress Recognition: 20.80%, all of which were lower than 60%. The positive response rates for six items were higher than 80%, including two items in Teamwork Climate, two items in Safety Climate, and two items not within any dimension. Ten items were lower than 60%: four items in Stress Recognition, four items in Recognition of Management, one item in Working Conditions, and one item not within any dimension. Descriptions of each item in the SAQ are shown in Table 7.

**Table 6:** The scores for healthcare workers in Beijing hospitals

Dimension	Mean (SD)	Positive response rate (%)
Teamwork climate	74.87 (18.23)	59.31^b^
Safety climate	73.82 (17.51)	54.09^b^
Job satisfaction	72.43 (22.50)	54.63^b^
Stress recognition	44.53 (28.70)	20.80^b^
Recognition of management	69.64 (19.68)	45.97^b^
Working condition	68.59 (20.09)	46.69^b^
Total scale	69.72 (15.47)	38.57^b^

Note: *SD* Standard deviation

^a^signifies the positive response rate is higher than 80%, ^b^signifies the positive response rate is lower than 60%

**Table 7:** The description of each item in Beijing hospitals

Item	Mean (SD)	Positive response rate (%)
Items belong to six dimensions		
Teamwork climate		
1. Nurse input is well received in this clinical area.	68.99(27.12)	65.70
2. In this clinical area, it is difficult to speak up if I perceive a problem with patient care. (R)	67.68(29.78)	63.89
3. Disagreements in this clinical area are resolved appropriately (i.e., not who is right, but what is best for the patient).	75.21(25.43)	74.50
4. I have the support I need from other personnel to care for patients.	74.49(26.79)	75.77
5. It is easy for personnel here to ask questions when there is something that they do not understand.	81.20(23.45)	83.29^a^
6. The physicians and nurses here work together as a well-coordinated team.	81.15(24.07)	82.91^a^
Safety climate		
7. I would feel safe being treated here as a patient.	80.24(22.87)	82.30^a^
8. Medical errors are handled appropriately in this clinical area.	81.32(21.86)	84.42^a^
9. I know the proper channels to direct questions regarding patient safety in this clinical area.	73.60(25.76)	70.51
10. I receive appropriate feedback about my performance.	66.48(28.23)	62.89
11. In this clinical area, it is difficult to discuss errors. (R)	68.69(28.15)	66.21
12. I am encouraged by my colleagues to report any patient safety concerns I may have.	69.45(25.35)	64.42
13. The culture in this clinical area makes it easy to learn from the errors of others.	76.66(22.95)	77.74
Job satisfaction		
15. I like my job.	68.13(26.97)	61.02
16. Working here is like being part of a large family.	77.41(24.57)	78.82
17. This is a good place to work.	72.80(27.74)	71.06
18. I am proud to work in this clinical area.	71.97(26.79)	66.51
19. Morale in this clinical area is high.	71.88(27.11)	67.49
Stress recognition		
20. When my workload becomes excessive, my performance is impaired. (R)	44.49(33.45)	32.97^b^
21. I am less effective at work when fatigued. (R)	41.97(32.74)	30.24^b^
22. I am more likely to make errors in tense or hostile situations. (R)	45.64(33.54)	35.93^b^
23. Fatigue impairs my performance during emergency situations (e.g. emergency resuscitation, seizure). (R)	46.00(33.86)	35.66^b^
Recognition of management		
24. Management supports my daily efforts.	69.16(23.79)	57.27^b^
25. Management doesn’t knowingly compromise patient safety.	76.43(24.6)	72.40
26. Management is doing a good job.	69.34(23.52)	57.09^b^
27. Problem personnel are dealt with constructively by our management.	66.63(23.83)	54.55^b^
28. I get adequate, timely information about events that might affect my work from Management.	65.96(23.94)	51.36^b^
Working condition		
29. The levels of staffing in this clinical area are sufficient to handle the number of patients.	56.94(32.27)	48.40^b^
30. This hospital does a good job of training new personnel.	72.41(24.77)	70.52
31. All the necessary information for diagnostic and therapeutic decisions is routinely available to me.	70.62(23.15)	69.13
32. Trainees in my discipline are adequately supervised.	74.40(21.55)	76.07
Items not belong to six dimensions		
14. My suggestions about safety would be acted upon if I expressed them to management.	61.81(28.48)	53.13^b^
33. I experience good collaboration with nurses in this clinical area.	82.33(19.74)	87.05^a^
34. I experience good collaboration with staff physicians in this clinical area.	80.46(20.6)	84.54^a^
35. I experience good collaboration with pharmacists in this clinical area.	75.82(22.75)	74.08
36. Communication breakdowns that lead to delays in delivery of care are common.(R)	65.22(31.67)	60.22

*Note*: *SD* Standard deviation

R indicates reverse-scored item

^a^signifies the positive response rate is higher than 80%

^b^signifies the positive response rate is lower than 60%

## Discussion

### The introduction and application of the SAQ in Chinese

The SAQ Short-Form version was introduced and applied in a safety culture survey of Beijing hospitals to evaluate the psychological characteristics and cross-cultural applicability of the SAQ in Chinese for the first time. The study showed that the SAQ in Chinese had very good internal consistency, similar to the English version [9] and versions in other languages [14, 16, 18, 19]; Cronbach’s alpha for the total scale was 0.945 and that for all dimensions was greater than 0.70. The validity analysis showed that the SAQ in Chinese had high content validity and good construct validity; most of the CFA indices indicated goodness for fit for the application of the SAQ model to the patient safety culture in Beijing.

The effective response rate for this survey was 87.9%, which was higher than the rates of similar studies in other countries (47.4%–69.4%) [9, 11, 14, 16, 19, 20]; the rate of missing items (0.06% to 1.38%) was also low [9, 14–16]. The success of the survey is attributable to several factors. First, all managers in the five hospitals supported the survey of patient safety culture. Second, we conducted the survey using on-the-spot questionnaires instead of email- or mail-based questionnaires because the delivery and recovery of the questionnaires through mail or email can result in lengthy collection times and questionnaire loss. Third, the introduction of the patient safety culture survey to the respondents was important for helping them fully understand the items. Fourth, the SAQ itself has favorable characteristics; it is a self-reported anonymous questionnaire, the number of items is moderate, the wording is easy to understand, and the scoring method is simple and easy to learn. Scoring with a machine-read card form is easy, which could facilitate an online survey platform for a large-scale survey such as in a city or for the entire country. The survey required a short amount of time (10–15 min) to complete. The above findings confirmed the acceptability of the SAQ in Chinese in practice.

We also recognize that the East and the West have different cultures, which can result in a different semantic understanding [38]. Although the translations were correct, non-equivalence of the concepts and their definitions may still exist [15], such as the word “appropriately” in “Disagreements in this clinical area are resolved appropriately”; “feedback” in “I receive appropriate feedback about my performance”; and “constructively,” in “Problem personnel are dealt with constructively by our management”. The evaluation criteria are obviously subjective. In addition, some items with reverse statements were difficult for the respondents to understand; thus, the answers may be variable and not consistent with the facts. Therefore, the respondents must carefully read the questions before providing the answers [18].

Therefore, the SAQ in Chinese requires further modification and improvement in theoretical research and in practice to serve as a safety culture evaluation tool that is suitable for respondents from Chinese cultural backgrounds.

### The analysis of stress recognition

Controversy exists regarding whether to keep the Stress Recognition dimension in the SAQ. In the correlation analysis of the structural validity, Stress Recognition showed a poor correlation with other dimensions, and similar results have been shown in previous studies on the SAQ in other languages [14, 15, 18–20].Similar studies have also recommended that Stress Recognition does not fit in the SAQ scale in terms of the theoretical construction of safety culture given its low explanatory power and that it could be removed when calculating the total score [20, 39]. Gallego et al. [40] indicated that Stress Recognition is different from other dimensions of the SAQ scale because it requires the evaluation of one’s behavior in the workplace, while the other dimensions of the SAQ focus on others’ behavior. In a study evaluating the effectiveness of safety culture improvement strategies using the SAQ, significant differences were seen before and after carrying out the improvement strategy, except for in the score for Stress Recognition [41].

We retained the dimension of Stress Recognition in the SAQ, as the developer’s intention was to assess frontline healthcare workers’ acknowledgement of how performance is influenced by stressors [9]; this dimension could remind them and their managers to pay attention to adverse conditions and take effective actions to enhance patient safety.

### The advantages and disadvantages of patient safety culture

The study showed that the safety culture was not sufficiently established at most hospitals in Beijing. The total mean score of the healthcare workers in the five hospitals was 69.72, and the respective scores of the six dimensions ranged from 44.53 to 74.87. Five dimensions (all except Stress Recognition) exhibited higher scores than baseline data from the United States, Britain, and Italy obtained in 2006 [9, 18], but slightly lower scores than the survey data reported by Taylor in 2011 [39]. The overall positive response rate was 38.57%; the positive response rates of the six dimensions ranged from 20.80% to 59.31% and were higher than the data obtained in Taiwan in 2010 [20]. However, all dimensions needed improvement to decrease the positive response rate to below the international standard (60%). Of the 36 items, six showed advantages, and ten items showed disadvantages.

Of the six dimensions, the scores and the positive response rates of Teamwork Climate were the highest, as in most studies [9, 14, 20, 39]. In the current complicated medical environment, healthcare providers have realized the importance of knowledge and complementary skills, resource sharing between team members, and establishing good cooperative relationships with colleagues to better manage conflict within the team with the clear target of ensuring patient safety. However, mutual trust between team members and the two-way communication capabilities in the department need to be improved, especially when perceiving a problem with patient care; speaking up is very important for patient safety, quality and efficiency in the patient treatment experience.

Compared to the other five dimensions in the study and this dimension in other studies [9, 14, 18, 39], the score and the positive response rates of Stress Recognition in the study were lower. The stress source (also called pressure source) is common in both China and other countries, but it is a far more serious issue in China because medical personnel’s job performance is affected to a great extent by insufficient human resources due to overwork, occupational stress, fatigue and other factors. Therefore, the medical environment and medical staff’s coping abilities and reaction intensity to stressful situations must be focused on and improved. Job burnout among medical staff is increasing, and satisfaction and loyalty are decreasing, which are important issues to address.

Four of five items in the Recognition of Management dimension showed disadvantages in the study, similar to the results in 2006 [9]. “*For effective safety management, leadership plays an important role at every level of management, ranging from team leaders to middle managers at a tactical level to top-level managers at the strategic level.*” [42]. Developing a hospital safety culture involves complicated system engineering; this culture must be constructed based on initiation from upper management, and leadership roles must be strengthened to drive the general organization’s cognitions and behavior changes toward safety recognition [43, 44].

In recent years, continued attention has been given to patient safety in domestic and foreign medical industries. Medical institutions in China are also committed to patient safety activities and have seen some advancements, while many patient safety projects focus on the improvement of unsafe factors in technology and procedures. Patient safety event reporting channels have been preliminarily established as part of the risk management system in hospitals in China since 2007, but the staff’s initiative and enthusiasm for reporting adverse events are affected by the punishment-based culture. Additionally, an effective safety culture has not been attained because appropriate feedback regarding the staff’s job performance cannot be obtained, and open discussion of errors and accidents in the department cannot be facilitated. Promotion activities related to safety culture in organizations are not being performed or effectively internalized [9], and the medical staff’s attitudes, cognition, abilities, and behavior patterns regarding patient safety still need to be improved.

A series of intervention strategies based on the principles of leadership, teamwork, and behavior changes have been designed and proven to be effective in improving both safety and patient outcomes in developed countries [45–54]; however, they are scarcely being applied in China. Executive Walk Rounds (EWRs) [47, 48] can enlist leadership to breakdown the significant barriers to discussion of human error in healthcare and help hospitals identify opportunities to improve care processes. They demonstrate the executives’ and the organization’s commitment to patient safety, and they may improve provider attitudes regarding safety-related issues. The Comprehensive Unit-based Safety Program (CUSP) [52] is a multifaceted strategy that includes elements of the science of safety training, safety hazard identification, senior executive partnership, learning from defects, and communication and teamwork. The Triad for Optimal Patient Safety Project (TOPS) [53] is expected to closely address teamwork within the unit and communication through an interdisciplinary team training intervention, involvement of a unit-based safety team to continue safety-focused teamwork, and establishment of a method for engaging patients within the multidisciplinary team. In addition, the establishment of a patient safety culture can be effectively combined with specific actions to enable safer patients to form a multiple-component organizational patient safety intervention project [54].

Due to the limitations of the study sample size, these results may not represent the overall data for Beijing hospitals, and the validation sample should be expanded in future research. The association between safety culture and patient outcomes also needs to be examined because the ultimate goal is to build a safe health system and reduce adverse medical events.

## Conclusions

As a valid assessment tool, the SAQ Short-Form in Chinese is suitable for the evaluation of safety culture in diverse clinical areas in Beijing hospitals. It demonstrates good psychometric properties, while some items require localization and further adaptation. This study provides baseline data for long-term continuous assessment and a reasonable basis for further targeted measures. The safety culture in most hospitals is not fully established and needs to be improved through intervention strategies. To insert the concept of “first, do no harm” into each unit and every operation code in the healthcare organization [55], we should accurately understand and grasp the connotation of a safety culture and its basic elements, highlight the important influence of human factors on the safety of patients, emphasize the important role of safety culture at every level of management, and constantly improve communication and cooperation within the team and between teams.

## Additional file

Additional file 1: The Safety Attitudes Questionnaire in Chinese. (DOCX 283 kb)

## Abbreviations

AGFI: Adjusted goodness-of-fit index
AIC: Akaike information criteria
CAIC: The Consistent Version of AIC
CFI: Comparative fit index
CN: Critical N
CVI: Content validity index
ECVI: Expected cross-validation index
FMAQ: Flight Management Attitude Questionnaire
GFI: Goodness-of-fit index
ICU: Intensive care Unit
I-CVI: Content validity index at the item level
IFI: Incremental fit index
NC: Normalized chi-square
NCP: Non-centrality parameter
NFI: Normed fit index
PGFI: Parsimony Goodness-of-Fit Index
PNFI: Parsimonious Normed Fit Index
RFI: Relative fit index
RMR: Root mean square residual
RMSEA: Root mean square error of approximation
SAQ: Safety Attitudes Questionnaire
S-CVI: Content validity index at the scale level
TLI: Tacker-Lewis index
