# Supplementary material for: The safety attitudes questionnaire in Chinese: psychometric properties and benchmarking data of the safety culture in Beijing hospitals
Source: BMC Health Serv Res. 2017 Aug 23;17:590. doi: 10.1186/s12913-017-2543-2 (PMC5568263; doi:10.1186/s12913-017-2543-2)
Supplement: Additional file 1: — The Safety Attitudes Questionnaire in Chinese. (DOCX 283 kb) [file 12913_2017_2543_MOESM1_ESM.docx]

安全态度问卷（SAQ量表）中文版

| **维度及定义** | **条目** |
| --- | --- |
| **团队合作**：同事间共同合作的感受。 | 1.本科室护理人员的意见可以充分被接受。  2.如果我感觉为患者提供医疗服务有困难时，在本科室不敢说出来。（反向问题）  3.本科室可以妥善地解决医疗工作中彼此间的意见分歧（如：不是谁对谁错，而是怎么做对患者最好）。  4.在为患者提供医疗服务时，我可以充分得到所需的支援。  5.在本科室工作如果有不清楚的地方可以很容易提问。  6.在本科室，医护人员是一个同心协力的工作团队。 |
| **安全氛围**：对于组织安全承诺的感受。 | 1.如果我是患者，我认为在本科室会得到十分安全的医疗服务。  2.在本科室医疗差错会被妥善地处理。  3.我知道有适当的渠道能直接反映与患者安全相关的问题。  4.我的工作表现可以得到适当的回馈。  5.在本科室并不容易讨论工作上的错误。（反向问题）  6.同事会鼓励我去报告任何与患者安全相关的疑虑。  7.本科室的文化氛围会让人很容易从别人的错误中汲取教训。 |
| **工作满意**：对于工作的积极性有正向的想法。 | 1.我非常喜欢我的工作。  2.在本科室工作就像是一个大家庭中的一份子。  3.本科室是一个很好的工作场所。  4.我以能够在这里工作为荣。  5.本科室的工作士气很高。 |
| **压力认知**：了解压力会影响工作成效。 | 1.工作负荷过重时，我的表现会变差。（反向问题）  2.疲倦时，我的工作会变得很没有效率。（反向问题）  3.在紧张或生气的情况下，我很容易犯错。（反向问题）  4.疲倦时，我应对紧急状况的能力会降低（如：急救复苏或处理癫痫发作等）。（反向问题） |
| **管理感知**：认同管理者的行事作风。 | 1.管理者能充分支持我日常的工作。  2.管理者不会刻意忽视患者安全。  3.管理者的工作做得很好。  4.管理者会以建设性的态度处理出现错误的人员。  5.对于可能会影响我工作的事件，可以从管理层得到充分且及时的信息。 |
| **工作条件**：对于工作环境和资源（如人力、设备）提供的感受。 | 1.本科室的人力配置足以处理当前工作量。  2.医院会做好新进人员的培训工作。  3.在做诊断与治疗决策时，我都可以获得所有必需的资讯。  4.在本专业领域的培训人员能得到适当的监督与指导。 |
